# Increasing thyroid cancer incidence in Canada, 1970–1996: time trends and age-period-cohort effects

**DOI:** 10.1054/bjoc.2001.2061

**Published:** 2001-09-01

**Authors:** S Liu, R Semenciw, A-M Ugnat, Y Mao

**Affiliations:** Health Surveillance and Epidemiology Division, Centre for Healthy Human Development, Health Canada, Ottawa; Surveillance and Risk Assessment Division, Centre for Chronic Disease Prevention and Control, Health Canada, Ottawa, Canada

**Keywords:** thyroid cancer, incidence, time trends, Poisson regression, Canada

## Abstract

We examined time trends in thyroid cancer incidence in Canada by age, time period and birth cohort between 1970 and 1996. Age-specific incidence rates by time period and birth cohort were calculated and age-period-cohort modelling used to estimate effects underlying the observed trends. Overall age-adjusted incidence rates of thyroid cancer doubled, from 3.3 and 1.1 per 100 000 in 1970–72 to 6.8 and 2.2 per 100 000 in 1994–96, among females and males respectively. Almost all the increase between 1970–72 and 1994–96 was due to papillary carcinoma of the thyroid. Age, birth cohort and period effects significantly improved the fit of the model for females, while age and birth cohort effects were significant determinants of the incidence among males. There were significant differences in the patterns/curvature for age, period and birth cohort effects between women and men. Our results suggest that the increases in thyroid cancer incidence in Canada may be associated with more intensive diagnostic activities and change in radiation exposure in childhood and adolescence. Temporal changes in reproductive factors among young women may explain some of the gender differences observed. © 2001 Cancer Research Campaign


*British Journal of Cancer* (2001) **85**(9), 1335–1339
© 2001 Cancer Research Campaign
doi: 10.1054/ bjoc.2001.2061, available online at http://www.idealibrary.com on IDEAL

### Increasing thyroid cancer incidence in Canada, 1970–1996: time trends and age-period-cohort effects

**S Liu[1], R Semenciw[2], A-M Ugnat[2] and Y Mao[2]**

[1]Health Surveillance and Epidemiology Division, Centre for Healthy Human Development, Health Canada, Ottawa; [2]Surveillance and Risk Assessment Division, Centre for Chronic Disease Prevention and Control, Health Canada, Ottawa, Canada

**Summary** We examined time trends in thyroid cancer incidence in Canada by age, time period and birth cohort between 1970 and 1996. Age-specific incidence rates by time period and birth cohort were calculated and age-period-cohort modelling used to estimate effects underlying the observed trends. Overall age-adjusted incidence rates of thyroid cancer doubled, from 3.3 and 1.1 per 100 000 in 1970–72 to 6.8 and 2.2 per 100 000 in 1994–96, among females and males respectively. Almost all the increase between 1970–72 and 1994–96 was due to papillary carcinoma of the thyroid. Age, birth cohort and period effects significantly improved the fit of the model for females, while age and birth cohort effects were significant determinants of the incidence among males. There were significant differences in the patterns/curvature for age, period and birth cohort effects between women and men. Our results suggest that the increases in thyroid cancer incidence in Canada may be associated with more intensive diagnostic activities and change in radiation exposure in childhood and adolescence. Temporal changes in reproductive factors among young women may explain some of the gender differences observed. © 2001 Cancer Research Campaign

**Keywords**: thyroid cancer; incidence; time trends; Poisson regression; Canada

Cancer of the thyroid is relatively uncommon, accounting for less than 2% of all cancers in Canada and worldwide (Coleman et al, 1993; Ron, 1995; National Cancer Institute of Canada, 2000). It consists of several morphologic types with distinct clinical and epidemiological features. Whereas papillary and follicular carcinomas occur mainly in the younger age groups, anaplastic tumours of thyroid are rare before 50 years of age. The female-to-male ratio is approximately 3:1 for papillary and follicular carcinomas, while medullary carcinoma occurs with almost equal frequency among males and females (Pettersson et al, 1991; Akslen et al, 1993; Ron, 1995; Zheng et al, 1996). Recent studies have shown that thyroid cancer incidence rates are steadily increasing in several countries including the United States (Spitz et al, 1988; Zheng et al, 1996), Sweden (Pettersson et al, 1991), Norway (Akslen et al, 1993) and Britain (Dos Santos Silva and Swerdlow, 1993). These increases have been accompanied by a change in the distribution of histologic types, however. Radiation is known to be an important risk factor (Ron et al, 1987; Spitz et al, 1988; Dos Santos Silva and Swerdlow, 1993; Inskip et al, 1995; Zheng et al, 1996; Mao et al, in press) but female hormonal and reproductive factors may also be relevant (Preston-Martin et al, 1987; Levi et al, 1993; Galanti et al, 1995; Paoff et al, 1995; Mack et al, 1999).

Zheng et al (1996) analysed cancer registration data for the last 60 years and identified a strong birth cohort effect underlying the observed increase in thyroid cancer incidence in both sexes in Connecticut, USA. This study also suggested that the introduction of radiation treatment for benign childhood conditions of the head and neck between the 1920s and the 1950s was largely responsible for the increasing temporal trends in thyroid cancer. We have therefore examined time trends for thyroid cancer incidence in Canada by age at diagnosis, time period and birth cohort between 1970 and 1996.

*Received 9 May 2001*
*Revised 9 June 2001*
*Accepted 10 June 2001*

*Correspondence to:* Y Mao

### MATERIALS AND METHODS

Thyroid cancer incidence data for 1992 to 1995 were obtained from the Canadian Cancer Registry (CCR) which replaced the National Cancer Incidence Reporting System (NCIRS) of Statistics Canada in 1992. Data for the province of Quebec were excluded because improved reporting procedures were not implemented there until 1981. The information regarding the CCR and the quality of Canadian cancer incidence data has been well documented (Band et al, 1993; Gaudette and Lee, 1997). In general, the cancer registry data included in this study are comparable and reliable, and 90% of thyroid cancers were pathologically confirmed in the period 1969–1973, and 95% in the period 1984–1988 (Band et al, 1993).

Information on histological classification of thyroid cancer was not consistently recorded by all provinces and territories in Canada until 1983, although Ontario, Saskatchewan and British Colombia collected such data even in earlier decades. The three provincial cancer registries used the Systematized Nomenclature of Pathology for histological classification until 1978, and all the records have been subsequently converted to the International Classification of Disease for Oncology (ICD-O). Based on the ICD-O first and second editions, thyroid cancer is coded as topography site codes 193 and C73, respectively. In this study, cases were subdivided into 3 broad histological groups: papillary, follicular and other specified or not otherwise specified (NOS) carcinomas.

We first contrasted average 3-year age-adjusted rates for the period 1970–72 with that for 1994–96 for both sexes. We then compared the rates in 4 broad age groups, i.e., 10–24, 25–44,

45–64 and 65–84 years. Secular trends in the incidence of thyroid cancer were evaluated through Poisson regression models using annual rates for all ages as well as for the described broad age groups. Correspondingly, the average annual percent changes (AAPC) during the study period were derived from the regression coefficients of those models. All age-adjusted incidence rates were calculated using direct standardization with the World Standard Population as the standard (Parkin et al, 1992).

In analyses integrating age at diagnosis, time period of diagnosis and birth cohort, age was grouped into 5-year intervals (15–19 years to 80–84 years). The period of diagnosis covered five equal 5-year intervals from 1972 to 1996. Corresponding to these age groups and time periods, a total of 18 overlapping 10-year birth cohorts (1887–1896 to 1972–1981, identified by the central year of birth from 1892 to 1977) were available for modelling of the incidence data.

A Poisson regression model was used to estimate the age, period and cohort effects. The model assumes that the number of cancer cases follows a Poisson distribution and the incidence rates are a multiplicative function of the included model parameters, making the logarithm of the rates an additive function of the parameters (Clayton and Schifflers, 1987a, 1987b; Holford, 1991, 1992). For example, the form of the age-period-cohort (APC) model was given by

$$\log (d_{ij}/p_{ij}) = \mu + \alpha_i + \beta_j + \gamma_k$$

where $d_{ij}$ denotes the number of the incident cases in the *i*th age group and *j*th period; $p_{ij}$, the population at risk in the *i*th age group and *j*th period; $\alpha_i$ the effect of the *i*th age group; $\beta_j$, the effect of the *j*th period category; and $\gamma_k$, the effect of the *k*th cohort category ($k = I - i + j$ when $i = 1,2,...,I$). Inherent in the three-factor APC model is the non-identifiability problem: parameters for age, period and cohort are not uniquely estimable because of the exact linear dependence of the regression variables (cohort = period – age) (Holford, 1992; Tarone and Chu, 1996). Although several methods for dealing with the non-identifiability problem have been proposed, there is no consensus in the literature as to which method is optimal. In our study, 3-factor APC models for the data were fitted by constraining the regression coefficients for the two extreme cohorts as zero, an approach commonly used in several previous studies (Tarone and Chu, 1996; Shahpar and Li, 1999; O'Callaghan et al, 2000). To compare the curvatures or the departure of the time trends in incidence between females and males in a single model (using an additional variable to define the gender), we introduced interaction terms between sex and the 3 time factors into the above general model (Holford, 1992; Liu et al, 2000). Changes in age, period and cohort effects were estimated along with the interaction terms.

Parameters of the models were estimated by means of the maximum likelihood method with SAS procedure GENMOD (release 6.12, SAS Institute Inc, Cary, NC 1997). The goodness-of-fit of models was evaluated using the deviance, defined to be twice the difference between the maximum achievable log likelihood and the log likelihood at the maximum likelihood estimates of the regression parameters. Specific effects (e.g., period effects) were tested by comparing the difference in deviance between models with and without a term for the effect.

### RESULTS

A total of 18 804 incident cases of thyroid cancer were registered in Canada excluding Quebec between 1970 and 1996. Of these, 13 975 (74.3%) were diagnosed in women and 4 829 (25.7%) in men. According to data for provinces of Ontario, Saskatchewan and British Columbia, 60% of 13 712 incident cases of thyroid cancer were classified as papillary carcinomas, 15% as follicular carcinoma and 25% as NOS.

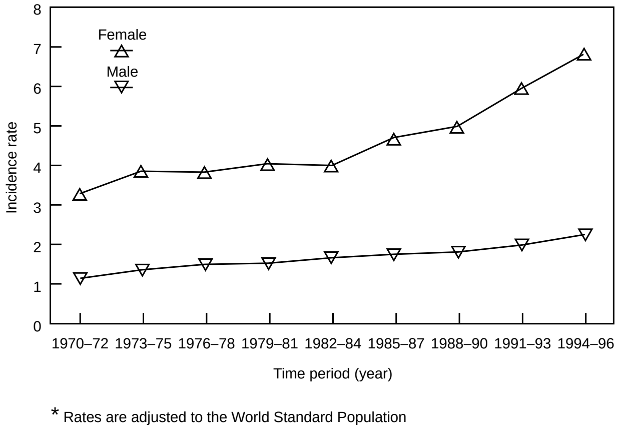

**Figure 1** Age-adjusted incidence rates of thyroid cancer by sex in Canada, 1970–72 to 1994–96, per 100 000

The overall age-adjusted rate among females and males increased substantially, from 3.5 and 1.1 per 100 000 population in 1970–72 to 6.7 and 2.2 per 100 000 population in 1994–96, respectively. The increasing trend among females appears to be more marked than that among males especially since the early 1980s (Figure 1), mostly due to an increase in papillary carcinoma which more than tripled between 1970–72 and 1994–96, from 1.4 to 5.4 per 100 000 among females and from 0.4 to 1.4 per 100 000 among males. Other types remained more or less stable (data available upon request).

The overall rate of increase in thyroid cancer among females was only slightly higher than that among males (AAPC: 3.5% vs 3.2%). However, the age-specific patterns of change were different across genders. For example, reproductive age-group women (age 25–44) experienced the most rapid increase (AAPC = 3.7%), while elderly women (age 65–84) had only a slight increase (AAPC = 0.7%) over the study period. In contrast, the differences in AAPCs between age groups were relatively smaller among men (Table 1).

The age-specific incidence rates by birth cohort are plotted in Figures 2 and 3 for females and males, respectively. The incidence showed an appreciable increase since the birth cohort of 1922 for both women and men, although the birth cohort effect was more evident among women. A high rate plateau was observed among those aged 30 to 64 years for women, while the incidence rates for men increased by age group, to the highest among the elderly aged 65 to 79 years (Figures 2 and 3).

Age-period-cohort models were fitted to the data for 1972 through 1996 for females and males separately. Table 2 summarizes the results of the modelling. Modelling of the data for females suggested that a full age-period-cohort model provided a significant improvement over either the age-cohort model or age-period model. The birth cohort effect was more pronounced than the period effect in terms of the ratio of the deviance and degrees of freedom. The full model was thus considered satisfactory for the time trends in thyroid cancer incidence among females. On the other hand, the age-cohort model fits the data for males well.

 *© 2001 Cancer Research Campaign*

**Table 1** Age-specific incidence rate of thyroid cancer (per 100 000 population) and average annual percent change (AAPC) in Canada excluding Quebec, 1970–72 to 1994–96[a]

| Age (year) | Female | | | Male | | |
|---|---|---|---|---|---|---|
| | 1970–72 | 1994–96 | AAPC[b] | 1970–72 | 1994–96 | AAPC[b] |
| 10–24 | 1.55 | 2.92 | 2.54** | 0.37 | 0.74 | 1.67* |
| 25–44 | 4.43 | 11.04 | 3.65** | 1.27 | 2.84 | 2.74** |
| 45–64 | 5.84 | 12.78 | 3.36** | 1.97 | 4.56 | 2.93** |
| 65–84 | 8.16 | 10.69 | 0.66* | 4.08 | 5.88 | 1.68** |
| All ages[a] | 3.26 | 6.82 | 3.50** | 1.12 | 2.23 | 3.15** |

*$P < 0.05$; **$P < 0.01$ [a]Rates were adjusted to the World Standard Population. [b]Trends were estimated by Poisson regression. See the Methods.

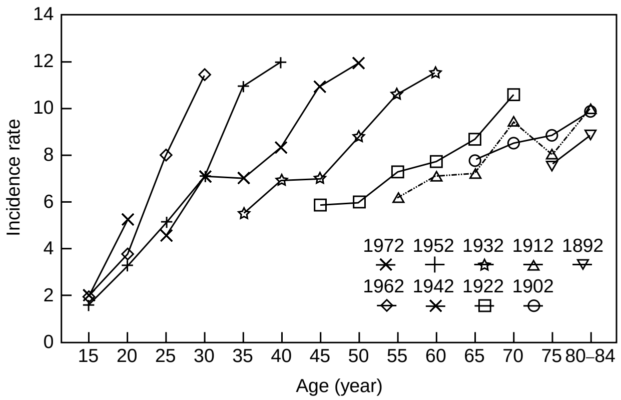

**Figure 2** Age-specific incidence rates of thyroid cancer by birth cohort among women in Canada, per 100 000

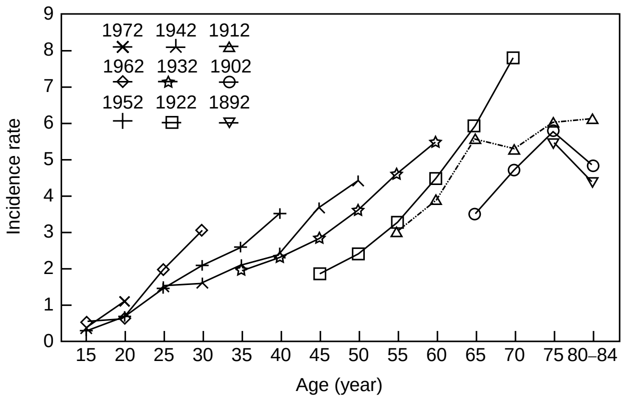

**Figure 3** Age-specific incidence rates of thyroid cancer by birth cohort among men in Canada, per 100 000

Neither the age-period model nor the full age-period-cohort model achieved a better fit. Therefore, the best-fitting model of the data for males showed a significant birth cohort effect in addition to an age effect.

All sex-curvature interactions with age, time period and birth cohort were statistically significant ($P < 0.05$ or $P < 0.01$), indicating that time trends among men differ significantly from women in terms of age at diagnosis, time period and birth cohort curvature effects. The contrast between the 'unique' trend lines obtained from fitting an age-period-cohort model showed different patterns between genders. For example, while the risk began to decrease around the age 55 among women, it instead increased sharply among men. On the other hand, the incidence trend rose steadily since the birth cohort of 1932 among women, while no clear changes were observed across the birth cohorts among men (Figure 4).

### DISCUSSION

Our results show that the overall age-adjusted incidence rates of thyroid cancer have been increasing in Canada among both females and males. The increase almost entirely comes from papillary carcinoma of the thyroid, with the greatest increase among young and middle-age women. This observation is similar to

**Table 2** Comparison and evaluation of age-period-cohort modelling of thyroid cancer incidence by sex in Canada excluding Quebec, 1972–1996

| Terms in model | Female | | | Male | | |
|---|---|---|---|---|---|---|
| | d.f[a] | Deviance[b] | *P* value[c] | d.f | Deviance | *P* value |
| Age | 56 | 695.9 | | 56 | 249.1 | |
| Age-period | 52 | 113.7 | 0.0001 | 52 | 105.7 | 0.0001 |
| Age-cohort | 39 | 68.2 | 0.0001 | 39 | 67.9 | 0.3279 |
| Age-period-cohort | 36 | 26.7 | | 36 | 64.5 | |

[a]d.f: degree of freedom. [b]Deviance from the standard Poisson model. [c]*P* values based on chi-square test refer to comparison between two-factor model with the full age-period-cohort model.

© 2001 Cancer Research Campaign

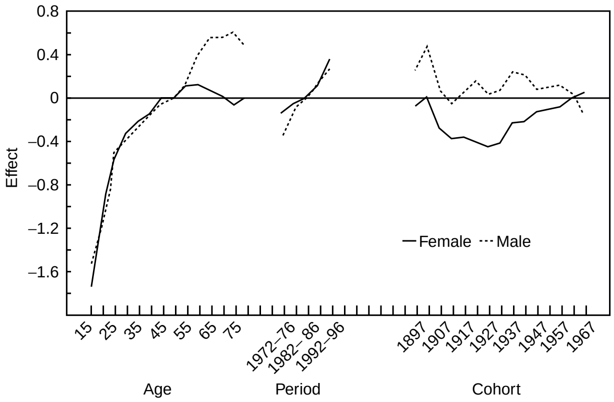

**Figure 4** Age, period and cohort effects on thyroid cancer incidence for females (solid line) and males (dashed line) estimated by a three-factor model with interaction terms for sex. The values for the first and last cohorts are not displayed (Tarone and Chu, 1996)

reports from the United States (Spitz et al, 1988; Zheng et al, 1996), Sweden (Pettersson et al, 1991), Norway (Akslen et al, 1993) and Britain (Dos Santos Silva and Swerdlow, 1993). Age-period-cohort modelling further shows that both birth cohort and period effects are responsible for the observed trends among females, while a birth cohort effect is the main determinant for the observed increase among males. More importantly, different sex patterns in terms of age, period and birth cohort curvatures were demonstrated, suggesting aetiologic heterogeneity between women and men in the pathogenesis of the disease (Figure 4).

Our results suggest that birth cohort effects are the major determinants of the observed trends in this population. Previous epidemiological studies have indicated that prior radiotherapy in childhood increases the risk of thyroid cancer (Boice, 1986; Ron et al, 1987; Spitz et al, 1988; Dos Santos Silva and Swerdlow, 1993; Inskip et al, 1995; Zheng et al, 1996; Mao et al, in press). Shore (1992) estimated that the risk of thyroid cancer associated with juvenile external irradiation is about 2.6 excess cancer/10 000 persons/year/gray. In Canada, ionizing radiation was used to treat children with benign conditions of the head and neck (e.g., enlarged thymus, tonsillitis and adenoids) between the 1930s and the 1960s. A population-based case–control study conducted in Connecticut, USA estimated that 9% of the disease in the population might be attributable to the prior radiotherapy to the head and neck in infancy (Ron et al, 1987).

Ionizing radiation is a main well-established risk factor for thyroid cancer (Shore et al, 1985; Franceschi and Vecchia, 1994; Ron, 1995). For the general population, diagnostic radiology may be the main source of exposure to ionizing radiation, and an increased risk associated with diagnostic X-rays is also biologically plausible (Shore et al, 1985; Franceschi and Vecchia, 1994). Furthermore, the thyroid gland may be included in the radiation field when either the upper body or the chest is exposed to X-rays. The susceptibility to radiation-induced thyroid cancer is the greatest for examinations occurring in childhood and adolescence (Boice, 1986; Ron et al, 1987; Spitz et al, 1988). Thus, our results suggest that the increasing trends in thyroid cancer incidence in Canada could be associated with change in radiation exposure in childhood and adolescence; nevertheless, the increases observed are larger than can be explained by the levels associated with current diagnostic procedures. A large prospective follow-up study (Inskip et al, 1995) found no tendency for case subjects to have experienced more X-ray procedures associated with a higher radiation dose to the thyroid gland. Diagnostic radiation also cannot satisfactorily explain the increases in the incidence observed only among women aged 25–64 years and the continued increase among recent birth cohorts.

Because of the marked female to male excess of well-differentiated thyroid cancers observed, a number of epidemiological studies have examined possible associations between hormones and other reproductive factors and the risk of thyroid cancer (Preston-Martin et al, 1987; Levi et al, 1993; Galanti et al, 1995; Paoff et al, 1995; Mack et al, 1999). Unlike the pattern observed for breast cancer, pregnancy and early menopause appear to enhance the risk of thyroid cancer (Preston-Martin et al, 1987; Levi et al, 1993). Furthermore, the association is more pronounced for the papillary type than the follicular type (Levi et al, 1993; Galanti et al, 1995). Among reproductive exposures, miscarriage or abortion, particularly in the first pregnancy is the most consistent risk factor (Preston-Martin et al, 1987; Levi et al, 1993; Ron, 1995). A recent pooled analysis (Negri et al, 1999) of original data from 14 case–control studies of thyroid cancer showed that there is a limited association with overall menstrual and reproductive factors, while miscarriage of the first pregnancy posts a high risk for developing papillary carcinoma (e.g., pooled OR 1.7 (95% CI 1.1–2.7)). In Canada, the therapeutic abortion rate for reproductive age women increased from 9.7 per 1000 in 1974 to 14.7 per 1000 in 1994, with the highest rates among young women aged 18–29 years (Health Statistics Division, 1996). However, the controversial findings regarding hormones and reproductive factors deserve further research in which confounders are better controlled.

More intensive diagnostic activity is another possible explanation for the increasing trends, given increased use of more sophisticated diagnostic methods (e.g., fine-needle biopsy, radio-isotope thyroid scanning) together with broader indications for the surgical removal of solitary nodules (Pettersson et al, 1991; Akslen, 1993; Dos Santos Silva and Swerdlow, 1993; Ron, 1995). Since papillary carcinomas have an indolent clinical course, intensive diagnostic activity may be leading to the discovery of occult papillary carcinomas. Corresponding to the rapid development in advanced histopathological techniques since the late 1970s, an increasing numbers of asymptomatic tumours of the thyroid may have been detected. In addition, histopathological misclassification cannot be ruled out in our data, as the introduction of new WHO histological criteria in 1974 had some impact on the changing temporal trends by histological subtype. It is likely, therefore, that an early detection artefact identified as a period effect in this study has played a role as well. Since that effect is only statistically significant in women, one may readily speculate that doctors may have increased their use of new diagnostic methods particularly in young patients and females. Nevertheless, the increase in papillary carcinoma and the underlying birth cohort effects cannot be explained entirely by increasing diagnosis of occult carcinomas or changes in classification.

Other risk factors such as radioactive fallout (Akslen et al, 1993; Dos Santos Silva and Swerdlow, 1993), iodine deficiency or iodine excess, endemic goiter (Pettersson et al, 1991; Franceschi and La Vecchia, 1994) and certain dietary factors appear to be unlikely to explain the overall temporal trend and the birth cohort pattern observed in this population. Since the causes of most thyroid cancer have yet to be discovered, the effect of these risk factors warrants further investigation.

 *© 2001 Cancer Research Campaign*

In conclusion, the steady increasing trends in the incidence of thyroid cancer during recent decades appears to be real. No single hypothesis regarding the associated risk factors can explain the rapidly increasing incidence. The birth cohort pattern observed for thyroid cancer incidence in the Canadian population suggests that the hypothesis that radiation exposure in childhood and adolescence is responsible for increasing risk of developing papillary carcinoma should be further investigated. Changes in female hormones and reproductive factors, particularly increased abortions or miscarriages, may partially explain the rapidly increased incidence among young and middle-aged women. Improved early detection may also have played a role, and should be further investigated. Other unrecognized aetiologic factors remain to be identified. The differential age, period and cohort effects provide clues to aetiologic heterogeneity in the pathogenesis of thyroid cancer between females and males.

### ACKNOWLEDGEMENTS

Data were provided to Health Canada from the Canadian Cancer Registry, formerly the National Cancer Incidence Reporting System, at Statistics Canada. The cooperation of the provincial and territorial cancer registries which supply the data to Statistics Canada is gratefully acknowledged.

### REFERENCES

Akslen LA, Haldorsen T, Thoresen SØ and Glattre E (1993) Incidence pattern of thyroid cancer in Norway: influence of birth cohort and time period. *Int J Cancer* **53**: 183–187

Band PR, Gaudette L, Hill GB, Holowaty EJ, Huchcroft SA, Johnston GM, Illing EM, Mao Y and Semenciw RM (1993) *The making of the Canadian cancer registry: Cancer incidence in Canada and it regions, 1969 to 1988* pp 16–21. Ottawa: Canadian Council of Cancer Registries

Boice JD Jr (1986) The danger of X-rays – real or apparent? (Editorial). *N Eng J Med* **315**: 828–830

Clayton D and Schifflers E (1987a) Models for temporal variation in cancer rates, I: Age-period and age-cohort models. *Stat Med* **6**: 449–467

Clayton D and Schifflers E (1987b) Models for temporal variation in cancer rates, II: Age-period-cohort models. *Stat Med* **6**: 469–481

Coleman MP, Estève J, Damiecki P, Arslan A and Renard H (1993) Trends in cancer incidence and mortality. (IARC Sci Publ no. 121): pp 673–704. Lyon: International Agency for Research on Cancer

Dos Santos Silva I and Swerdlow AJ (1993) Thyroid cancer epidemiology in England and Wales: time trends and geographical distribution. *Br J Cancer* **67**: 330–340

Franceschi S and La Vecchia C (1994) Thyroid carcinoma. In: Trends in cancer incidence and mortality, Vol. 19/20, Doll R. (ed) pp 393–422. Fraumeni JF, Muir CS. Cold Spring Harbor Laboratory Press, New York

Galanti MR, Lambe M, Ekbom A, Sparen P and Pettersson B (1995) Parity and risk of thyroid cancer: a nested case-control study of a nationwide Sweden cohort. *Cancer Causes and Control* **6**: 37–44

Gaudette L and Lee L (1997) Cancer incidence in Canada, 1969–1993. Ottawa; Ministry of Industry (catalogue 82–566-XPB)

Health Statistics Division (1996) Therapeutic Abortions 1994. Catalogue No. 82–219-XPB Statistics Canada. Ottawa

Holford TR (1991) Understanding the effects of age, period, and cohort on incidence and mortality rates. *Annu Rev Public Health* **12**: 425–457

Holford TR (1992) Analysing the effects of age, period, and cohort on incidence and mortality rates. *Stat Meth Med Res* **1**: 317–337

Holford TR, Zheng T, Mayne ST and Mckay LA (1992) Time trends of Non-Hodgkin's Lymphoma: Are the real? What do they mean? *Cancer Research* **52**(suppl) : 5443–5446

Inskip PD, Ekbom A, Galanti MR, Grimelius L and Boice JD Jr (1995) Medical diagnostic X rays and thyroid cancer. *J Natl Cancer Inst* **87**: 1613–1621

Levi F, Franceschi S, Gulie C, Negri E and La Vecchia C (1993) Female thyroid cancer: the role of reproductive and hormonal factors in Switzerland. *Oncology* **50**: 309–315

Liu S, Semenciw R, Waters C, Wen SW, Mery LS and Mao Y (2000) Clues to the etiologic heterogeneity of testicular seminomas and non-seminomas: Time trends and age-period-cohort effects. *Int J Epidemiol* **29**: 826–831

Mack WJ, Preston-Martin S, Bernstein L, Qian D and Xiang M (1999) Reproductive and hormonal risk factors for thyroid cancer in Los Angeles County females. *Cancer Epidemiol Biomarker Prev* **8**: 991–997

Mao Y, Ugnat A-M, Hill G, Fry R, Kreiger N and Fincham S. Ionizing radiation exposure as a risk factor for thyroid cancer. Cancer Prevention and Control (in press)

National Cancer Institute of Canada (2000) Canadian Cancer Statistics 2000 pp 13–41. Toronto

Negri E, Maso LD, Ron E, Vecchia CL, Mark SD and Preston-Martin S (1999) A pooled analysis of case-control studies of thyroid cancer: II. Menstrual and reproductive factors. *Cancer Causes and Control* **10**: 143–155

O'Callaghan FK, Osmond C and Marthn CN (2000) Trends in epilepsy mortality in England and Wales and the United States, 1950–1994. *Am J Epidemiol* **151**: 182–189

Paoff K, Preston-Martin S, Mack WJ and Monroe K (1995) A case-control study of maternal risk factors for thyroid cancer in young women (California, United States). *Cancer Causes and Control* **6**: 389–397

Parkin DM, Muir CS, Whelan SL, Gao YT, Ferlay J and Powell J (1992) (ed) Cancer incidence in five continents. Vol. VI, pp 865–870 Lyon: International Agency for Research on Cancer (IARC Sci Publ no.120)

Pettersson B, Adami HO, Wilander E and Coleman MP (1991) Trends in thyroid cancer incidence in Sweden, 1958–1981, by histo-pathological type. *Int J Cancer* **48**: 28–33

Preston-Martin S, Bernstein L, Pike MC, Maldonado AA and Henderson BE (1987) Thyroid cancer among young women related to prior thyroid disease and pregnancy history. *Br J Cancer* **55**: 191–195

Ron E (1995) Thyroid cancer In: Peckham M (ed). Pinedo H, Veronesi U, Oxford Textbook of Oncology. Vol 2, pp 1000–1021. Oxford University Press, Oxford

Ron E, Kleinerman RA, Boice JD Jr, Livolsi VA, Flannery JT and Fraumeni JF Jr (1987) A population-based case-control study of thyroid cancer. *J Natl Cancer Inst* **79**: 1–12

Shahpar C and Li G (1999) Homicide mortality in the United States, 1935–1994: age, period, and cohort effects. *Am J Epidemiol* **150**: 1213–1222

Shore RE, Woodard E, Hildreth N, Dvoretsky P, Hempelmann L and Pasternack B (1985) Thyroid tumors following thymus irradiation. *J Natl Cancer Inst* **74**: 1177–1184

Shore RR (1992) Issues and epidemiological evidence regarding radiation-induced thyroid cancer. *Rad Res* **131**: 98–111

Spitz MR, Sider JG, Katz RL, Pollack ES and Newell GR (1988) Ethnic patterns of thyroid cancer incidence in the United States, 1973–1981. *Int J Cancer* **42**: 549–553

Tarone RE and Chu K (1996) Evaluation of birth cohort patterns in population disease rates. *Am J Epidemiol* **143**: 85–91

Zheng T, Holford TR. Chen Y, Ma JZ, Flannery J and Liu W (1996) Time trend and age-period-cohort effect on incidence of thyroid cancer in Connecticut, 1935–1992. *Int J Cancer* **67**: 504–509

*© 2001 Cancer Research Campaign*
